# Modeling Human Heart Development and Congenital Defects Using Organoids: How Close Are We?

**DOI:** 10.3390/jcdd9050125

**Published:** 2022-04-21

**Authors:** Shan Jiang, Wei Feng, Cindy Chang, Guang Li

**Affiliations:** Department of Developmental Biology, School of Medicine, University of Pittsburgh, Pittsburgh, PA 15201, USA; shine278987826@163.com (S.J.); weifeng@pitt.edu (W.F.); cyc15@pitt.edu (C.C.)

**Keywords:** heart development, congenital heart defect, organoid, anatomical structure, cardiac lineage, marker gene, signaling pathway

## Abstract

The emergence of human-induced Pluripotent Stem Cells (hiPSCs) has dramatically improved our understanding of human developmental processes under normal and diseased conditions. The hiPSCs have been differentiated into various tissue-specific cells in vitro, and the advancement in three-dimensional (3D) culture has provided a possibility to generate those cells in an in vivo-like environment. Tissues with 3D structures can be generated using different approaches such as self-assembled organoids and tissue-engineering methods, such as bioprinting. We are interested in studying the self-assembled organoids differentiated from hiPSCs, as they have the potential to recapitulate the in vivo developmental process and be used to model human development and congenital defects. Organoids of tissues such as those of the intestine and brain were developed many years ago, but heart organoids were not reported until recently. In this review, we will compare the heart organoids with the in vivo hearts to understand the anatomical structures we still lack in the organoids. Specifically, we will compare the development of main heart structures, focusing on their marker genes and regulatory signaling pathways.

## 1. Introduction

To model the in vivo developmental processes, 3D cell cultures were developed and categorized into different subtypes such as embryoid body (EB), gastruloid, spheroid, and organoid. EBs are aggregated differentiating embryonic stem cells (ESCs) or iPSCs. The cells in EBs are usually at early differentiation stages and do not organize into specific spatial patterns. In the cardiac stem cell field, EBs are used to generate lineage-specific cell types such as cardiomyocytes [1]. Gastruloids consist of three germ layers and are used to model the embryo gastrulation process. In theory, gastruloids can develop into an entire embryo if an appropriate differentiation environment is provided; in a recent study, a differentiation condition was designed to enable the cultured mouse embryos to develop to the hindlimb formation stage [2,3,4]. The spheroid is an aggregate of differentiated cell types or cancer cells. These cells do not self-assemble into the anatomical patterns observed in vivo and can be used to study the interactions among different cell types. Lastly, the organoid is a complicated cell aggregate in which the differentiated cells mostly belong to a specific organ and self-assemble into the in vivo-like anatomic patterns. Tissue-specific organoids, such as the cerebrum, liver, lung, and kidney, can be made with iPSCs or progenitor cells [5,6]. They usually have similar morphology and cell-type interactions to the in vivo organs, making organoids advantageous over other 3D models [7]. However, as the emergence of multi-lineage organoids and certain organoids can only recapitulate part of the organ-like features, the distinctions between the different types of 3D cell cultures are becoming less clear.

Multiple hiPSC-derived heart organoid systems were recently reported and claimed to have developed in vivo-like heart features. The heart is the first organ to develop in humans, and its defects affect about 1% of births [8]. Heart development originates from mesodermal cells, which later develop into cardiac progenitors in the two heart fields. First heart field (FHF) cells develop into the primitive heart tube, and second heart field (SHF) progenitors contribute to heart-tube elongation from both poles [9,10]. Meanwhile, the heart tube undergoes rightward looping, giving rise to the four-chambered heart. The heart chamber wall consists of three tissue layers: epicardium, myocardium, and endocardium. The heart also has other structures besides the chambers, including the inflow tract (IFT), the atrial ventricular canal (AVC), and the outflow tract (OFT) at the early stages, which contribute to the development of the chambers, valves, septum, and large vessels at later stages. The normal development of these structures is essential for generating a functional heart; if this process goes awry, it can cause congenital heart defects (CHDs) [10].

Although mouse models are highly valuable in studying certain CHDs such as hypoplastic left heart syndrome [11], many CHDs still do not have helpful animal models due to the potential genetic and pathological differences between species. Human-induced Pluripotent Stem Cells (hiPSCs) have a significant advantage in modeling these diseases, as the iPSCs can be directly reprogrammed from patient somatic cells, therefore preserving the genetic background of the patients. To model CHDs, the 3D differentiation condition (e.g., organoids) is essential as it can potentially develop features like those seen in the in vivo hearts. Besides modeling normal heart developmental processes and CHDs, heart organoids also have the potential to be used for drug screenings. In this review, we will compare the developmental processes of in vivo hearts and heart organoids to learn the potential ways to improve organoid systems further, with a goal to generate organoids with structures and functions like in vivo hearts.

## 2. Heart fields Formation

**Figure 1:**
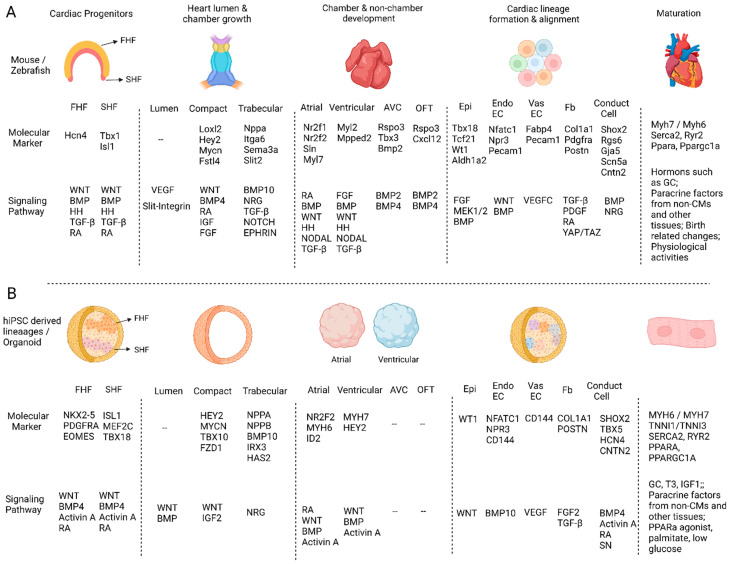
The comparison of molecular markers and regulatory signaling pathways in in vivo heart and human heart organoid development. (**A**) The major events during in vivo heart development and their molecular markers and regulatory signaling pathways. (**B**) The molecular markers and signaling pathways that have been reported to be important in regulating human iPSC-derived cardiac cell or cardiac organoid differentiations. “--” means non-applicable or no related studies have been reported.

During mouse heart development, the two heart fields are specified sequentially and express marker genes such as *Hcn4* (FHF), *Isl1*, and *Tbx1* (SHF) [12,13]. The FHF originates from the lateral plate mesoderm and mainly contributes to linear heart-tube formation. FHF formation is induced by signals from the adjacent ectoderm, endoderm, embryonic midline, and posterior regions. Bone morphogenetic protein (BMP), Fibroblast growth factor (FGF), Transforming growth factor β (TGFβ), and WNT signaling pathways were reported to be involved in this process [14,15]. The SHF is derived from the pharyngeal mesoderm and contributes to heart development from both inflow and outflow poles after the linear heart-tube stage. Additionally, the SHF is specified by signals from the surrounding pharyngeal endoderm and neural crest cells. While SHF progenitor cell survival, proliferation, and deployment were found to be dependent upon WNT, BMP, Hedgehog (HH), and FGF signaling [15,16], the posterior limit of SHF was reported to be determined by retinoic acid (RA) [17]. Furthermore, TGF-β, HH, and FGF were reported to be important in zebrafish SHF progenitor cell proliferation and differentiation [18] (Figure 1A).

The FHF and SHF were also discovered to develop in mouse 3D culture systems. Andersen et al. verified FHF/SHF-like cells in their mouse iPSC-derived organoids by showing similarities with embryonic FHF/SHF cells in their gene expression and differentiation potentials. They also found that the Bmp/Smad pathway and the Smad-independent BMP/WNT pathway specified FHF and SHF progenitors [12], respectively. Recently, Rossi et al. described mouse embryonic stem cells (mESCs)-derived gastruloids that were found to have cells expressing the FHF/SHF markers and had a spatial distribution like the in vivo progenitors [19]. Additionally, Lewis-Israeli et al. generated heart organoids from hiPSCs presenting characteristics of both heart fields in the same organoid. They identified *NKX2-5, PDGFRA*, and *EOMES* expression in the FHF progenitors and *ISL1*, *MEF2C*, and *TBX18* expression in SHF cells (Figure 1B). These progenitors were described to respectively develop into left and right ventricular CMs based on the expression of *HAND1* and *HAND2* [20].

Next, it will be interesting to analyze the detailed structures in the organoid heart fields, as the FHF and SHF in mouse embryos are specified into small segments through the differential expression of HOX genes and RA signaling, and each segment respectively develops into related heart anatomical structures such as atrial and ventricular chambers [21]. Furthermore, these organoid heart fields do not seem to differentiate sequentially or form the specific spatial patterns seen in the heart fields in embryos, whose SHF locates dorsal and medial to the FHF to progressively contribute cells to both poles of the linear heart tube [10]. With further detailed analysis and appropriate manipulations, we expect the organoid heart fields to develop in a similar temporal and spatial manner as seen in embryonic heart field development.

## 3. Heart Lumen Development

Through genetic screening in Drosophila, the heart tube lumen formation was found to be regulated by a Slit-Integrin signaling pathway, which regulates actin cytoskeleton alignment to promote cardiac cell polarization in lumen development [22,23]. In mice, live imaging analysis revealed that the heart lumen developed from a split of two endocardial endothelial cell (EndoEC) layers at the cardiac crescent stage. It also found that cell rounding was unlikely to initiate lumen formation as the cardiac crescent cells are still columnar when the lumen begins to develop [24]. As part of the heart lumen developmental process, aorta lumen propagation initiates between stages 1S and 3S (E8.0), developing from adjacent endothelial cell (EC) contact after EC shape has changed. This process is regulated by VE-Cadherin and VEGF-A [25]. In humans, the heart lumen develops from the fusion of two endocardial tubes, each of which has a hollow lumen derived from the cardiogenic cords [26].

A recent study reported the induction of heart chamber-like structures in human heart organoids and found that Wnt-BMP signaling and transcription factor *HAND1* were both critical in this process [27]. Further time-course analysis of organoid formation found that the lumen appeared after 2.5–3.5 days of differentiation at the cardiac mesoderm stage, which is earlier than when the mouse heart lumen develops in the cardiac crescent stage. Additionally, the study found that low WNT and Activin A levels can induce chamber formation with a partial inner lining of EndoECs. However, lumen formation does not seem to rely on the EndoECs, as the lumen can still form when the EndoECs developed on the outer organoid surface after VEGF treatment [27]. Lewis-Israeli et al. and our study also generated organoids with chamber-like structures, and these chambers also developed independently from the EndoECs [20,28]. As chamber formation in the current organoids does not go through the same process as in vivo heart lumen development, there is limited value in modeling human heart lumen formation under normal and diseased conditions using organoid cultures. However, heart organoids may still be valuable in studying other aspects of heart chamber development, such as heart pumping and looping.

## 4. Compact and Trabecular Myocardium Growth

Heart chamber growth was thought to balloon out from the looped hearts segmentally. The ventricular and atrial chambers were found to respectively expand from the linear heart tube on ventral and dorsal sides [29,30] and the expanded chambers to develop into two types of myocardium, with the compact myocardium on the outer surface and trabecular myocardium close to the lumen to increase cardiac output and oxygen uptake at early embryonic stages [31,32]. The CMs in compact myocardium highly express *Loxl2*, *Hey2*, *Mycn*, and *Fstl4*, while the CMs in trabecular myocardium express *Nppa*, *Itga6*, *Sema3a*, and *Slit2* [32]. Compact and trabecular myocardium development was shown to be differentially regulated by signals from the epicardium and the endocardium [33,34,35]. Epicardium-derived signals such as BMP4, FGF, WNT, IGF, and RA were reported to promote CM proliferation in compact myocardium [35,36,37], and endocardium signals such as NOTCH, Neuregulin, Ephrin, and TGF-β were reported to promote trabecular myocardium development [31,38,39]. Furthermore, some signaling molecules expressed in the myocardium, such as BMP10, were also found to regulate the trabecular myocardium development [40] (Figure 1A).

To generate compact CMs from hiPSCs, WNT and IGF2 were added to the ventricular CM differentiation system on day 10 [41]. The CMs were shown to express typical compact myocardium marker genes such as *HEY2*, *MYCN*, *TBX10*, and *FZD1*. In contrast, the addition of Neuregulin to the differentiation system at day 10 can specify CMs into trabecular CMs expressing trabecular myocardium genes such as *NPPA*, *NPPB*, *BMP10*, *IRX3*, and *HAS2* (Figure 1B). Similarly, the co-culture of EndoEC with CMs can promote the development of trabecular CMs, as EndoECs were known to be able to secrete Neuregulin in mice and zebrafish [42]. Next, it will be interesting to test if other EndoEC-derived growth factors such as TGF-β and NOTCH can also induce trabecular CM fate and if epicardium-derived factors such as RA, BMP, WNT, and FGF can promote compact CM development. Additionally, and most importantly, a test will be needed to determine if these factors can be applied locally to generate heart organoids with compact and trabecular myocardium at correct anatomical locations.

## 5. Heart Structure Development

The early stages of mouse heart development consist of the formation of the four chambers (left and right atrial; left and right ventricular) and two non-chamber structures—the atrial ventricular canal (AVC) and the outflow tract (OFT). While AVC at later developmental stages contributes to the development of the septum and atrioventricular valves, including the tricuspid and mitral valves, the OFT contributes to the formation of large vessels (aorta and pulmonary artery) and the semilunar valves, including the aortic and pulmonary valves [43]. The atrial CMs highly express *Nr2f1*, *Nr2f2*, *Sln*, and *Myl7*, while the ventricular CMs express *Myl2* and *Mpped2*. Furthermore, while the left and right ventricular CMs differentially express *Pcsk6*, the left and right atrial CMs highly express *Pitx2* and *Shox2*, respectively. In contrast, the AVC CMs express *Rspo3*, *Tbx3,* and *Bmp2*, and the OFT CMs express *Rspo3* and *Cxcl12* [32,44] (Figure 1A).

Atrial lineage specification is regulated by RA signaling in multiple species [45,46,47], while early dorsal-ventral patterning signals such as FGF and BMP also differentially promote atrial and ventricular lineage development in zebrafish [48]. As the left and right ventricular CMs develop from different heart fields, their lineage formation is primarily regulated by the heart field specification signals previously mentioned when discussing heart fields’ formation. The AVC and OFT share a structure named the endocardial cushion, which is induced by the interaction of BMP signaling, including BMP2 and BMP4 in myocardium and BMPR1A in EndoECs. Mouse endocardial cushion cells express marker genes such as *Twist1*, *Msx1*, and *Snail*. Endocardial cushion cells need to go through an endothelial-to-mesenchymal transition (EndoMT) process regulated by multiple signaling pathways, such as TGFβ, WNT/β-catenin, HIPPO, and NOTCH, to develop into valve cells [49].

Atrial and Ventricular CMs were found to co-exist in heart organoids but did not display in vivo-like spatial domains. RA signaling had been used to promote atrial CM lineage in monolayer and EB-based hiPSC differentiation, and the atrial and ventricular CM progenitors were distinguished based on the expression of *CD235A* and *RALDH2* [50,51]. We have also generated heart organoids with atrial or ventricular identities by adding (+) or omitting (−) RA at the cardiac mesoderm stage and found that the RA+ and RA- heart organoids had distinct membrane action potentials and Ca^2+^ transient activities. The chamber identity of these cells was further confirmed with immunofluorescence staining for chamber-specific marker genes such as *MYH7*, *HEY2* (ventricular), *NR2F2*, *MYH6*, and *ID2* (atrial) (Figure 1B). We also performed single-cell mRNA sequencing (scRNA-seq) and random forest-based zone classification to analyze their cell identities systematically [28]. These analyses consistently support that the CMs in RA- heart organoids preferentially develop into ventricular CMs, while the CMs in RA+ organoids are more likely to develop into atrial CMs [28].

Interestingly, our study found that *MYL2* is a robust ventricular CM marker gene in human fetal hearts but is barely expressed in the organoid ventricular CMs differentiated from the hiPSC line “WTC”. Considering that WTC and its derived transgenic lines have been broadly used in the cardiac stem cell field, we have investigated *MYL2* expression in WTC-derived CMs from multiple labs based on their scRNA-seq results. We found that while *MYL2* was barely detected in the WTC-derived CMs before differentiation day 30 in several studies, it was expressed in other hiPSC line-derived CMs with the same differentiation conditions [52,53,54]. However, there are some exceptions where *MYL2* was found to be expressed in the WTC-derived CMs on day 30 in one study and day 90 in another. Both studies generated the CMs using a monolayer with small molecules protocol [53,55] (Table 1). The cause of the differences in gene expression across the studies is still a mystery to us, but it will be important to investigate whether this expression variation also exists in other genes and other cell lines.

HiPSC-derived valve mesenchymal cells are known to be induced from EndoECs through EndoMT, and FGF, VEGF, and BMP signaling was found to be important in this process. After the treatment of FGF8 and VEGF, pre-valvular endocardial ECs with the expression of *CD31* were induced, and through further comparative analysis with scRNA-seq, those cells were found to be similar to the mouse AVC endocardium at E9.0 (embryonic day 9). Those cells can undergo EMT after BMP2 treatment and express valvular interstitial cell genes such as *MSX1*, *SMAD6*, *SOX9*, *SLUG*, *CADHERIN 11*, *N-CADHERIN*, and *PERIOSTIN* [56]. Another study found that BMP10 is vital to EndoEC induction. Through BMP10 and bFGF treatment, an Nkx2-5^+^ CD31^+^ EndoEC population was identified and found to express *NFATC1*, *NPR3*, *GATA4*, and *GATA5*. After BMP2 and TGF-β treatment, these EndoECs were able to undergo EndoMT and develop into valvular interstitial-like cells (VICs) expressing *NR4A2*, *PRRX2*, and *TIMP3* [42]. Next, it will be interesting to adapt this process to organoid systems.

## 6. The Anatomical Pattern of Cardiac Cells

In mammalians, the heart chambers consist of three tissue layers: epicardium, endocardium, and myocardium. The epicardium develops from proepicardium, and its developmental process was found to be regulated by FGF, MEK1/2, and myocardium-derived BMP signaling [57]. Epicardial cells in the epicardium were characterized with *Tbx18*, *Tcf21*, *Wt1*, and *Aldh1a2* expression and the cells were able to undergo EMT to develop into smooth muscle cells (SMCs) and cardiac fibroblasts (CFs). This process was reported to be regulated by many signaling pathways such as TGF-β, PDGF, RA, and Yap/Taz [58], and the CFs were shown to express multiple marker genes, such as *Col1a1*, *Postn*, and *Pdgfra* [44]. The EndoEC expresses marker genes *Pecam1*, *Nfatc1*, and *Npr3,* and was found to develop from the Flk1+ multipotent cardiovascular progenitors in the FHF [59] and vascular endothelial progenitors in the SHF [60]. These cells have the plasticity to develop into many cell types such as cushion mesenchymal cells, vascular ECs, and vascular mural cells [61], and cell development is regulated by cardiac progenitor cell determination signals such as Wnt and Bmp at an early stage and is influenced by the myocardium-derived signals at later stages. The coronary vascular ECs express *Pecam1* and *Fabp4* [62] and its cells at the dorsal and ventral sides were respectively derived from sinus venosus and endocardium. Additionally, sinus venous-derived Vas EC development was proved to be promoted by VEGFC [63], and a recent study found that the position of VasECs was guided by chemokine signals, such as Slit2, from the epicardium-derived cells (EPDCs) [58] (Figure 1A).

The mammalian heart conduction system consists of the Sinoatrial (SA) node, the Atrioventricular (AV) node, the His bundle, and Purkinje fibers. ScRNA-seq analysis of developing mouse conduction cells revealed that SA node cells express *Shox2*, *Rgs6*, and *Smoc2*; AV node and His bundle cells express *Kcne1*, *Tbx5*, and *Rgs6*; and the Purkinje fiber cells express *Gja5*, *Scn5a*, *Etv1*, and *Nkx2-5* [64]. Lineage tracing studies demonstrated that conduction cells were mainly derived from cardiomyocytes [65]. Furthermore, the development of conduction cells is regulated by the signaling pathways essential for the AVC and trabecular myocardium development, such as BMP and NRG [65].

The organoids reported by Hofbauer et al. contained CMs, ECs, and Fb-like cells [27]. Interestingly, they found that low WNT and Activin A led to a high proportion of CMs with VEGF-A expression, which can further direct the specific patterning of ECs at the inner part of the cavity structure to resemble the specific patterning of in vivo EndoECs. These ECs expressed the EndoEC markers *NFATC1* and *NPR3*, and their transcriptomic profiles were comparable with human umbilical vein endothelial cells (HUVECs) and human cardiac microvascular endothelial cells (HCMECs). Consistently, Lewis-Israeli et al. also identified CMs, ECs, and Fbs in their organoids [20]. We also developed heart organoids with the three major cardiac cell types [28]. Interestingly, we found that the ECs were mostly EndoECs. As the percentage of EndoECs was small, they did not cover the entire organoid lumen surface. A recent study reported that EndoECs could be induced by BMP10 in an EB system [42]. It will be interesting to test if this factor can also improve the EndoEC differentiation efficiency in heart organoids. Vascular ECs were also observed in heart organoids but did not form vascular-like structures. This was probably caused by the low EC differentiation efficiency and the lack of an adequate environment, such as hypoxia and blood flow, to maintain their identities. In order to develop vascularized organoids, multiple methods were proposed, including (1) co-culture with ECs, (2) co-differentiation with mesodermal progenitor cells, (3) mechanical stimulation, and 4) in vivo transplantation into a vascular enriched locus such as the kidney capsule [66,67,68].

Significantly few epicardial cells were observed in the heart organoids without specific proepicardial induction. To incorporate epicardium into their organoids, Hofbauer et al. generated spheroids with epicardial cells and further fused them with heart organoids. They found that the epicardial cells migrated into the heart organoids and underwent EMT to differentiate into EPDCs [27]. Additionally, Lewis-Israeli et al. induced proepicardial lineage in organoids by introducing CHIR (a WNT activator) at a relatively late differentiation stage (day 7). They found that a short period of CHIR treatment was sufficient to induce a layer of epicardium on the organoid outer surface, and the ratio of CMs to epicardial cells was like in vivo (60–65% cardiomyocytes:10–20% epicardial cells) [20].

Conduction cells have not been identified in heart organoids, but these cell types have been reported to be differentiated in EB and monolayer systems. SA nodal-like cells were isolated in hiPSC-derived atrial cardiomyocyte populations based on the lack of NKX2-5 expression, and their differentiation was found to be enhanced with BMP4 and TGF-β antagonist treatment. Additionally, RA was revealed to enhance the pacemaker phenotype of the SA-like cells [69]. To generate cardiac Purkinje fiber cells, a small molecule screening experiment was carried out, and sodium nitroprusside (SN) was identified to be able to convert CM into Purkinje cells by activating cyclic AMP signaling [70] (Figure 1B). Next, it will be interesting to test if conduction cells can be induced in heart organoids by treating them with related growth factors or small molecules at specific stages.

## 7. Tissue Maturation

The mammalian heart undergoes maturation from fetal to adult stages to become a fully functional organ. Cardiomyocyte maturation is associated with changes in gene expression, morphology, and functional readouts. The matured CMs are characterized by the expression of distinct myofibril gene isoforms and metabolism pathway genes, the formation of organized sarcomere structures, T-tubules, polyploidization, and the development of improved sarcomere contraction and action potentials [71]. Along the developmental progression, multiple events were thought to promote CM maturation. At fetal stage, non-CMs, such as fibroblasts, appearing at E13.5-E14.5 in mice were reported to secrete paracrine factors to regulate CM proliferation and maturation [72,73,74]; hormones such as glucocorticoids are synthesized and transported to the heart starting around E14.5 to promote heart maturation [75]. The heart switches from hypoxia to a normoxia environment at the neonatal stage, with oxygen as a known maturation factor. At the same time, the heart also changes its energy source from glucose to lipid [76]. Additionally, the development of other tissues and physiological functions (contraction, blood flow) can also contribute to heart maturation [77,78].

The matured heart organoid is essential to modeling many features in heart function, and several methods have been developed to improve this aspect. The treatment of hiPSC-derived 3D heart microtissue with three hormones (Thyroid, Dexamethasone, and IGF-1) was found to improve tissue maturation, which was proved by the development of enhanced electrophysiological properties, and adult CM-like sarcomere structure and gene expression profile [79]. Funakoshi et al. further improved the method by using a combination of PPARa agonist, palmitate, dexamethasone, thyroid, and low glucose to treat heart EBs. Their method was found to be able to mature the ventricular compact and atrial CMs, as these treated cells developed aligned sarcomere structures, enhanced contraction ability, a high mitochondria mass, and fatty acid-based metabolism [41] (Figure 1B). Meanwhile, the co-culture of CM, EC, and FB in 3D tissue was found to enhance the CM maturation [80]. Additionally, multi-lineage organoids, such as those with gut and heart lineages, had more matured CMs than those with heart lineages only [19,81,82]. The in vivo heart environment was also reported to be important in promoting CM maturation: when hiPSC-derived CMs were transplanted into neonatal and adult rat hearts, they were found to gain partially matured myofibrils [83].

## 8. Application of the Heart Organoids

Although heart organoids still lack many features observed in in vivo hearts, they have been successfully applied in modeling several heart development and injury-related processes. We used organoids to model a congenital heart defect named Ebstein’s anomaly (EA) [28], which is characterized by an atrialized right ventricular chamber. To model the defect, we first generated isogenic hiPSC lines carrying an EA-associated point mutation on NKX2-5. We then specified the cell lines into atrial and ventricular organoids by adding or omitting RA in the differentiation process, respectively. We found the diseased organoids from the conditions without RA treatment to have higher beating rates (the atrialized ventricular phenotype) than the control organoids in the same differentiation condition. This was similar to what was observed in EA patients. Further on, we utilized voltage recording and scRNA-seq to analyze these organoids and found the diseased cells from the ventricular differentiation condition to consistently have atrial CM-like features, a phenocopy of the defects in patients. In another study, Lewis-Israeli et al. used heart organoids to model pregestational diabetes-induced CHDs by treating them with glucose and insulin. They found that the glucose/insulin-treated organoids displayed irregular action potential shapes, impaired glycolysis and oxygen consumption, and disturbed distribution of mitochondria and lipid droplets, suggesting successful modeling of cardiomyopathy caused by oxidative stress and metabolic disorders [20]. Hofbauer et al. used heart organoids to study the heart injury process by treating their heart organoids with cryoinjury. Their study observed the recruitment of COL1A1+ fibroblast and accumulation of fibronectin at the injury site, mimicking an early aspect of regenerative and fibrotic responses [27].

## 9. Conclusions

Here, we have compared the developmental process in embryonic hearts and heart organoids by focusing on their anatomical structures, marker genes, and regulatory signaling pathways. As several important pieces are still missing in the current human heart organoids, to precisely model in vivo heart developmental processes, these pieces need to be added or induced in situ. In particular, we emphasized the importance of the temporal and spatial coordination of developmental events such as heart field specification, heart lumen development, and cardiac lineage differentiation. However, considering that most heart organoid systems were only reported recently, it is possible to significantly improve them in the coming years. Although the current organoids have their limitations, they have been successfully used to model several heart development and injury processes such as atrial/ventricular lineage specification defects and fibrosis after heart injury.

Compared to the in vivo hearts with four chambers and multiple non-chamber structures, the current heart organoids have only one chamber with mainly atrial or ventricular lineages. In the future, it will be crucial to generate organoids with lineages from specific chambers, such as the atrial and ventricular left and right sides. Additionally, four-chambered organoids need to be induced or assembled using chamber-specific organoids, as this feature will be necessary for modeling many heart developmental and physiological processes such as heart looping and heart blood circulation.

To model the early heart developmental events using organoids, we also need to consider the effects of biomechanical forces on cardiac morphogenesis carefully. Biomechanical forces have been found to play an important role in multiple heart developmental processes such as heart looping, myocardium trabeculation, chamber septation, and valve formation [84,85]. Applying appropriate forces to the cultured organoids will be crucial to inducing each staged morphogenesis.

Cardiac cell lineages can be induced in situ in organoids or added later after differentiation, as demonstrated in the gastrointestinal organoids incorporated with cells from three different germ layers [86]. To generate heart organoids like the in vivo hearts, the identity of induced cells, including their cell type, organ specificity, and maturation need to be carefully characterized. These cells can be analyzed with scRNA-seq and further compared to scRNA-seq data of primary cells at fetal and adult stages. Multiple human cell atlases with cells from all major organs at different stages have been reported and can serve as the standard dataset for comparative analysis [87,88].

The spatial pattern of each cell lineage is essential to generating functional heart organoids. However, a standard map with the precise anatomical location of each cell type is still missing. Emerging spatial transcriptomics and tissue clearing methods can potentially provide a solution for this [89,90]. Once a detailed heart map is generated, the differentiated cells can, in theory, be accurately printed out using a bioprinter [91]. For instance, using a FRESH 3D bioprinting method, Bliley et al. [92] printed a linear heart tube and Mirdamadi et al. printed a full-size model of the human heart with hiPSC-derived CMs [93]. Besides bioprinting, the human ventricle-like cardiac chambers have also been generated by embedding CMs with a nanofibrous scaffold or collagen-based extracellular matrix hydrogel [94,95]. Although these chambers were shown to have chamber-level contractile function and physiological features, they do not contain all the main cardiac lineages such as fibroblasts and endothelial cells. Alternatively, the different lineages can also be induced in situ by controlling the concentration of growth factors in a temporally and spatially pattern. Future work from multidisciplines including developmental biology, stem cell biology, molecular biology, biomaterials, and bioengineering will be essential to generate functional four-chambered hearts in a dish.

## Figures and Tables

**Table 1:** The *MYL2* expression in WTC parent and derived hiPSC lines in different studies.

	MYL2 Expression	Stage	Differentiation Protocol
Feng et al. (BioRxiv, https://doi.org/10.1101/2020.12.24.424346)	Barely expressed	Day 30	Monolayer with small molecules, Organoid with small molecules
Paige et al. (PMID: 33074758)	Abundant expression	Day 30	Monolayer with small molecules
Grancharova et al. (PMID: 34349150)	Barely expressed	Day 26	Monolayer with a combination of cytokines and small molecules
Grancharova et al. (PMID: 34349150)	Abundant expression	Day 90	Monolayer with small molecules
Friedman et al. (PMID: 30290179)	Barely expressed	Day 30	Monolayer with small molecules

## Data Availability

The single cell RNA sequencing data was downloaded from GEO under the accession numbers GSE106118 (Feng et al.) and GSE146763 (Paige et al.), ArrayExpress database under the accession number E-MTAB-6268 (Friedman et al.), and the allencell database with the link: https://open.quiltdata.com/b/allencell/packages/aics/wtc11_hipsc_cardiomyocyte_scrnaseq_d0_to_d90 (accessed on 8 March 2022) (Grancharova et al.).
